# 2-{[2-(2-Hy­droxy-5-meth­oxy­benzyl­idene)hydrazin-1-yl­idene]meth­yl}-4-meth­oxy­phenol

**DOI:** 10.1107/S1600536813034636

**Published:** 2014-01-11

**Authors:** Muhammad Taha, Syed Adnan Ali Shah, Sadia Sultan, Nor Hadiani Ismail, Sammer Yousuf

**Affiliations:** a Atta-ur-Rahman Institute for Natural Product Discovery, Universiti Teknologi MARA (UiTM), Puncak Alam Campus, 42300 Bandar Puncak Alam, Selangor D. E., Malaysia; b Faculty of Applied Science, Universiti Teknologi MARA (UiTM), 40450 Shah Alam, Malaysia; c Faculty of Pharmacy, Universiti Teknologi MARA (UiTM), Puncak Alam Campus, 42300 Bandar Puncak Alam, Selangor Darul Ehsan, Malaysia; d H.E.J. Research Institute of Chemistry, International Center for Chemical and Biological Sciences, University of Karachi, Karachi 75270, Pakistan

## Abstract

The title phenyl­hydrazine derivative, C_16_H_16_N_2_O_4_, has a crystallographically imposed centre of symmetry. Except for the methyl group, all non-H atoms are almost coplanar (r.m.s. deviation = 0.0095 Å). Intra­molecular O—H⋯N hydrogen bonds are observed, generating *S*(6) graph-set ring motifs.

## Related literature   

For applications and the biological activity of phenyl­hydrazine derivatives, see: Khan *et al.* (2013 ▶); Patel *et al.* (1984 ▶); Taha, Baharudin *et al.* (2013 ▶); Taha, Ismail *et al.* (2013 ▶); Khan, Shah *et al.* (2012 ▶); Khan, Taha *et al.* (2012 ▶). For structures of related compounds, see: Taha *et al.* (2012 ▶); Kargar *et al.* (2012 ▶); Zhang *et al.* (2008 ▶).
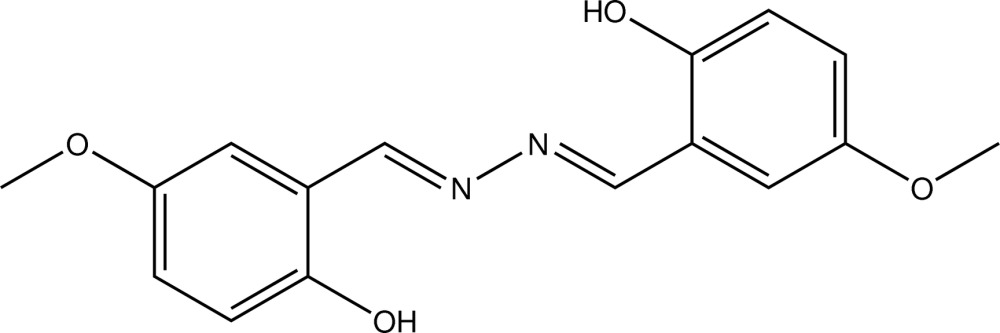


## Experimental   

### 

#### Crystal data  

C_16_H_16_N_2_O_4_
*M* *_r_* = 300.31
Monoclinic, 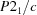

*a* = 6.7132 (4) Å
*b* = 15.9369 (10) Å
*c* = 6.8022 (4) Å
β = 91.192 (2)°
*V* = 727.59 (8) Å^3^
*Z* = 2
Mo *K*α radiation
μ = 0.10 mm^−1^
*T* = 273 K
0.58 × 0.22 × 0.17 mm

#### Data collection  

Bruker SMART APEX CCD area-detector diffractometer
Absorption correction: multi-scan (*SADABS*; Bruker, 2000 ▶) *T*_min_ = 0.944, *T*_max_ = 0.983
4243 measured reflections
1327 independent reflections
1093 reflections with *I* > 2σ(*I*)
*R*_int_ = 0.016

#### Refinement  

*R*[*F* ^2^ > 2σ(*F* ^2^)] = 0.034
*wR*(*F* ^2^) = 0.101
*S* = 1.06
1327 reflections
106 parameters
H atoms treated by a mixture of independent and constrained refinement
Δρ_max_ = 0.14 e Å^−3^
Δρ_min_ = −0.12 e Å^−3^

### 

Data collection: *SMART* (Bruker, 2000 ▶); cell refinement: *SAINT* (Bruker, 2000 ▶); data reduction: *SAINT*; program(s) used to solve structure: *SHELXS97* (Sheldrick, 2008 ▶); program(s) used to refine structure: *SHELXL97* (Sheldrick, 2008 ▶); molecular graphics: *SHELXTL* (Sheldrick, 2008 ▶); software used to prepare material for publication: *SHELXTL*, *PARST* (Nardelli, 1995 ▶) and *PLATON* (Spek, 2009 ▶).

## Supplementary Material

Crystal structure: contains datablock(s) global, I. DOI: 10.1107/S1600536813034636/rz5090sup1.cif

Structure factors: contains datablock(s) I. DOI: 10.1107/S1600536813034636/rz5090Isup2.hkl

Supporting information file. DOI: 10.1107/S1600536813034636/rz5090Isup3.cml

CCDC reference:

Additional supporting information:  crystallographic information; 3D view; checkCIF report

## Figures and Tables

**Table 1:** Hydrogen-bond geometry (Å, °)

*D*—H⋯*A*	*D*—H	H⋯*A*	*D*⋯*A*	*D*—H⋯*A*
O2—H2⋯N1	0.94 (2)	1.82 (2)	2.6451 (16)	145.0 (18)

**Fig. 1.**
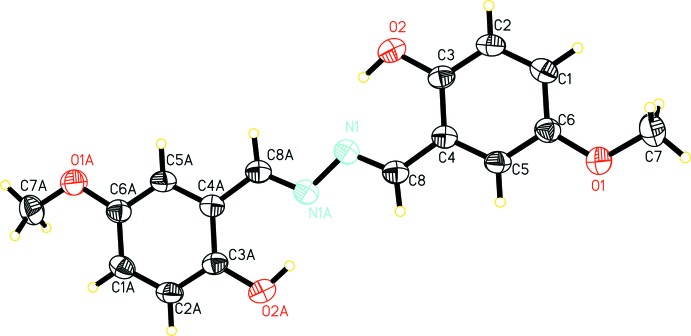
The molecular structure of the title compound with displacement ellipsoids drawn at the 30% probability level. Symmetry code: (A) -x, 1-y, 1-z.

**Fig. 2.**
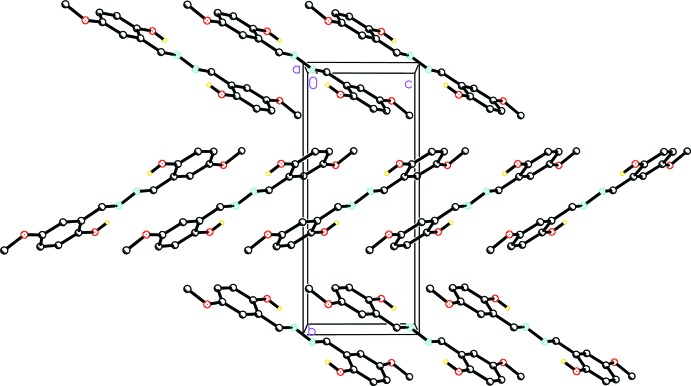
Crystal packing of the title compound viewed down the a axis.

## supplementary crystallographic information

### 1. Comment

Hydrazone derivatives constitute an important class of biologically active drugs (Khan, Shah *et al.*, 2012). In particular, heterocyclic compounds containing the hydrazone moiety are known to possess excellent activity against *Mycobacterium tuberculosis* H37Rv (Patel *et al.*, 1984). Recently the antioxidant, antiglycating, and antileishmanial activities of different hydrazine derivatives has also been reported by our group (Taha, Baharudin *et al.*, 2013; Taha, Ismail *et al.*, 2013; Khan, Taha *et al.*, 2012; Khan *et al.*, 2013).

The title compound (Fig. 1) has crystallographically imposed centre of symmetry, the inversion centre lying midway along the N—N bond. Except for the methyl group, all non-hydrogen atoms in the asymmetric unit are coplanar with an r.m.s. deviation of 0.0095 Å. The carbon atom of the methyl group is displaced by 0.1806 (17) Å from this plane. All bond angles and lengths are found to be normal and similar to those observed in structurally related compounds (Taha *et al.*, 2012; Kargar *et al.*, 2012; Zhang *et al.*, 2008). The molecular configuration is stabilized by a pair of intramolecular O2—H2···N1 hydrogen interactions generating *S*(6) graph-set ring motifs. The crystal structure is stabilized only by van der Waals contacts (Fig. 2).

### 2. Experimental

The title compound was synthesized by refluxing a mixture of 2-hydroxy-5-methoxybenzaldehyde (2 mmol, 0.304 g) and hydrazine hydrate (55%, 2 ml) in methanol with a catalytical amount of acetic acid for 1 hour. After completion of the reaction, the solvent was evaporated by vacuum to afford the crude product which was further recrystallized in methanol to obtain needle-like crystals suitable for X-ray analysis (yield 88%, 0.524 g).

### 3. Refinement

H atoms were positioned geometrically with C—H = 0.93-0.96 Å and constrained to ride on their parent atoms with *U*_iso_(H) = 1.2*U*_eq_(C) or 1.5*U*_eq_(C) for methyl H atoms. A rotating group model was applied to the methyl group. The hydroxy H atom was located in a difference Fourier map and refined isotropically.

### Crystal data

**Table:**

C_16_H_16_N_2_O_4_	*F*(000) = 316
*M**_r_* = 300.31	*D*_x_ = 1.371 Mg m^−^^3^
Monoclinic, *P*2_1_/*c*	Mo *K*α radiation, λ = 0.71073 Å
Hall symbol: -P 2ybc	Cell parameters from 1747 reflections
*a* = 6.7132 (4) Å	θ = 2.6–27.0°
*b* = 15.9369 (10) Å	µ = 0.10 mm^−^^1^
*c* = 6.8022 (4) Å	*T* = 273 K
β = 91.192 (2)°	Needle, yellow
*V* = 727.59 (8) Å^3^	0.58 × 0.22 × 0.17 mm
*Z* = 2	

### Data collection

**Table:**

Bruker SMART APEX CCD area-detector diffractometer	1327 independent reflections
Radiation source: fine-focus sealed tube	1093 reflections with *I* > 2σ(*I*)
Graphite monochromator	*R*_int_ = 0.016
ω scan	θ_max_ = 25.5°, θ_min_ = 2.6°
Absorption correction: multi-scan (*SADABS*; Bruker, 2000)	*h* = −8→7
*T*_min_ = 0.944, *T*_max_ = 0.983	*k* = −18→19
4243 measured reflections	*l* = −7→8

### Refinement

**Table:**

Refinement on *F*^2^	Secondary atom site location: difference Fourier map
Least-squares matrix: full	Hydrogen site location: inferred from neighbouring sites
*R*[*F*^2^ > 2σ(*F*^2^)] = 0.034	H atoms treated by a mixture of independent and constrained refinement
*wR*(*F*^2^) = 0.101	*w* = 1/[σ^2^(*F*_o_^2^) + (0.0505*P*)^2^ + 0.0718*P*] where *P* = (*F*_o_^2^ + 2*F*_c_^2^)/3
*S* = 1.06	(Δ/σ)_max_ < 0.001
1327 reflections	Δρ_max_ = 0.14 e Å^−^^3^
106 parameters	Δρ_min_ = −0.12 e Å^−^^3^
0 restraints	Extinction correction: *SHELXTL* (Sheldrick, 2008), Fc^*^=kFc[1+0.001xFc^2^λ^3^/sin(2θ)]^-1/4^
Primary atom site location: structure-invariant direct methods	Extinction coefficient: 0.031 (6)

### Special details

**Table:**

Geometry. All e.s.d.'s (except the e.s.d. in the dihedral angle between two l.s. planes) are estimated using the full covariance matrix. The cell e.s.d.'s are taken into account individually in the estimation of e.s.d.'s in distances, angles and torsion angles; correlations between e.s.d.'s in cell parameters are only used when they are defined by crystal symmetry. An approximate (isotropic) treatment of cell e.s.d.'s is used for estimating e.s.d.'s involving l.s. planes.
Refinement. Refinement of *F*^2^ against ALL reflections. The weighted *R*-factor *wR* and goodness of fit *S* are based on *F*^2^, conventional *R*-factors *R* are based on *F*, with *F* set to zero for negative *F*^2^. The threshold expression of *F*^2^ > σ(*F*^2^) is used only for calculating *R*-factors(gt) *etc*. and is not relevant to the choice of reflections for refinement. *R*-factors based on *F*^2^ are statistically about twice as large as those based on *F*, and *R*- factors based on ALL data will be even larger.

### Fractional atomic coordinates and isotropic or equivalent isotropic displacement parameters (Å^2^)

**Table:**

	*x*	*y*	*z*	*U*_iso_*/*U*_eq_	
O1	0.45722 (17)	0.62697 (7)	−0.27087 (17)	0.0865 (4)	
O2	−0.21947 (15)	0.62524 (8)	0.17505 (19)	0.0767 (4)	
H2	−0.199 (3)	0.5903 (15)	0.284 (3)	0.118 (8)*	
N1	−0.01775 (16)	0.52608 (7)	0.41923 (16)	0.0588 (3)	
C1	0.1157 (2)	0.67416 (8)	−0.2207 (2)	0.0626 (4)	
H1C	0.1136	0.7055	−0.3361	0.075*	
C2	−0.0501 (2)	0.67158 (9)	−0.1050 (2)	0.0636 (4)	
H2B	−0.1632	0.7015	−0.1436	0.076*	
C3	−0.0522 (2)	0.62571 (8)	0.0666 (2)	0.0562 (4)	
C4	0.12003 (18)	0.58088 (7)	0.12530 (19)	0.0518 (3)	
C5	0.28603 (19)	0.58474 (8)	0.0070 (2)	0.0585 (4)	
H5A	0.4008	0.5559	0.0453	0.070*	
C6	0.2857 (2)	0.63003 (8)	−0.1651 (2)	0.0594 (4)	
C7	0.4762 (3)	0.68202 (11)	−0.4318 (2)	0.0842 (5)	
H7A	0.6065	0.6762	−0.4856	0.126*	
H7B	0.4577	0.7388	−0.3884	0.126*	
H7C	0.3773	0.6686	−0.5307	0.126*	
C8	0.12864 (19)	0.53066 (8)	0.3019 (2)	0.0564 (4)	
H8A	0.2442	0.5006	0.3314	0.068*	

### Atomic displacement parameters (Å^2^)

**Table:**

	*U*^11^	*U*^22^	*U*^33^	*U*^12^	*U*^13^	*U*^23^
O1	0.0721 (7)	0.0981 (9)	0.0899 (8)	0.0108 (6)	0.0178 (6)	0.0323 (6)
O2	0.0593 (6)	0.0892 (8)	0.0818 (8)	0.0171 (5)	0.0052 (5)	0.0019 (6)
N1	0.0624 (7)	0.0524 (6)	0.0614 (7)	−0.0025 (5)	−0.0015 (5)	0.0012 (5)
C1	0.0741 (9)	0.0526 (8)	0.0607 (9)	0.0041 (6)	−0.0104 (7)	0.0025 (6)
C2	0.0633 (8)	0.0590 (8)	0.0678 (9)	0.0147 (6)	−0.0135 (7)	−0.0034 (6)
C3	0.0529 (7)	0.0509 (7)	0.0647 (9)	0.0039 (5)	−0.0051 (6)	−0.0089 (6)
C4	0.0528 (7)	0.0427 (6)	0.0597 (8)	−0.0012 (5)	−0.0049 (6)	−0.0028 (5)
C5	0.0524 (7)	0.0519 (7)	0.0711 (9)	0.0051 (5)	−0.0038 (6)	0.0063 (6)
C6	0.0591 (8)	0.0535 (7)	0.0654 (9)	−0.0007 (6)	−0.0010 (6)	0.0039 (6)
C7	0.0942 (12)	0.0909 (12)	0.0679 (10)	−0.0096 (9)	0.0087 (9)	0.0141 (8)
C8	0.0549 (7)	0.0473 (7)	0.0666 (9)	−0.0004 (5)	−0.0045 (6)	−0.0006 (6)

### Geometric parameters (Å, º)

**Table:**

O1—C6	1.3713 (17)	C2—H2B	0.9300
O1—C7	1.4105 (18)	C3—C4	1.4101 (18)
O2—C3	1.3563 (16)	C4—C5	1.3894 (17)
O2—H2	0.93 (2)	C4—C8	1.4436 (18)
N1—C8	1.2809 (15)	C5—C6	1.3752 (19)
N1—N1^i^	1.394 (2)	C5—H5A	0.9300
C1—C2	1.377 (2)	C7—H7A	0.9600
C1—C6	1.386 (2)	C7—H7B	0.9600
C1—H1C	0.9300	C7—H7C	0.9600
C2—C3	1.377 (2)	C8—H8A	0.9300
			
C6—O1—C7	118.38 (12)	C6—C5—C4	121.95 (12)
C3—O2—H2	109.0 (13)	C6—C5—H5A	119.0
C8—N1—N1^i^	113.84 (13)	C4—C5—H5A	119.0
C2—C1—C6	119.96 (14)	O1—C6—C5	116.20 (12)
C2—C1—H1C	120.0	O1—C6—C1	124.77 (13)
C6—C1—H1C	120.0	C5—C6—C1	119.03 (13)
C1—C2—C3	121.54 (12)	O1—C7—H7A	109.5
C1—C2—H2B	119.2	O1—C7—H7B	109.5
C3—C2—H2B	119.2	H7A—C7—H7B	109.5
O2—C3—C2	119.17 (12)	O1—C7—H7C	109.5
O2—C3—C4	121.76 (13)	H7A—C7—H7C	109.5
C2—C3—C4	119.07 (13)	H7B—C7—H7C	109.5
C5—C4—C3	118.45 (12)	N1—C8—C4	122.15 (12)
C5—C4—C8	119.20 (11)	N1—C8—H8A	118.9
C3—C4—C8	122.35 (12)	C4—C8—H8A	118.9
			
C6—C1—C2—C3	−0.1 (2)	C7—O1—C6—C5	−170.46 (14)
C1—C2—C3—O2	−179.95 (12)	C7—O1—C6—C1	10.2 (2)
C1—C2—C3—C4	0.4 (2)	C4—C5—C6—O1	−178.37 (12)
O2—C3—C4—C5	−179.65 (11)	C4—C5—C6—C1	1.0 (2)
C2—C3—C4—C5	−0.03 (18)	C2—C1—C6—O1	178.72 (13)
O2—C3—C4—C8	1.09 (19)	C2—C1—C6—C5	−0.6 (2)
C2—C3—C4—C8	−179.29 (12)	N1^i^—N1—C8—C4	179.45 (12)
C3—C4—C5—C6	−0.68 (19)	C5—C4—C8—N1	178.39 (12)
C8—C4—C5—C6	178.60 (12)	C3—C4—C8—N1	−2.36 (19)

Symmetry code: (i) −*x*, −*y*+1, −*z*+1.

### Hydrogen-bond geometry (Å, º)

**Table:**

*D*—H···*A*	*D*—H	H···*A*	*D*···*A*	*D*—H···*A*
O2—H2···N1	0.94 (2)	1.82 (2)	2.6451 (16)	145.0 (18)
